# Assessing the Impact of High Body Mass Index (BMI) on the Efficacy of Assisted Reproductive Technologies (ART) in Saudi Women: A Cross-Sectional Study Examining Ovarian Reserve and Treatment Outcomes

**DOI:** 10.7759/cureus.46706

**Published:** 2023-10-09

**Authors:** Abdulsalam Aleid, Manal Y Alturaifi, Ruba I Alharbi, Fatema Saleh, Lubna H Alomari, Raghad Hazazi, Hala A Sindi, Rufaida A Ahmed, Abbas Al Mutair

**Affiliations:** 1 Neurosurgery, King Faisal University, Al-Ahsa, SAU; 2 College of Medicine, King Faisal University, Al-Ahsa, SAU; 3 General Medicine, Batterjee Medical College, Jeddah, SAU; 4 College of Medicine, Taibah University, Medina, SAU; 5 Physical Therapy, Johns Hopkins Aramco Healthcare, Dhahran, SAU; 6 College of Medicine, King Abdulaziz University Faculty of Medicine, Jeddah, SAU; 7 Obstetrics and Gynecology, Maternity and Children Hospital, Al-Ahsa, SAU; 8 Research Center, Almoosa Specialist Hospital, Al-Ahsa, SAU

**Keywords:** ivf & icsi, saudi women, live births, treatment outcomes, cycle numbers, art, ovarian, pcos, high bmi

## Abstract

Introduction: The global surge in high body mass index (BMI) and obesity has led to various health complications. While numerous studies have shown that obesity disrupts female fertility, the specific effects of obesity on the success rate of assisted reproductive technology (ART) treatments in Saudi women have been less explored. This study aimed to delve into this gap, especially focusing on the correlation between BMI, ovarian reserve parameters, and ART outcomes among Saudi women.

Methods: A cross-sectional study was carried out from January to August 2023, concentrating on Saudi women aged 18 and above who underwent ART treatments for infertility. A total of 1071 women participated, with 155 completing an online survey and 916 responding through a hard copy from several Saudi hospitals. The data encompassed demographics, medical history, anthropometric details, ovarian reserve parameters, and ART results. For the analysis, Statistical Package for the Social Sciences (IBM SPSS Statistics for Windows, IBM Corp., Version 28.0, Armonk, NY) was utilized, applying descriptive statistics, the Chi-square test, and a linear regression model to discern connections between BMI, participant characteristics, and ART outcomes. A p-value of less than 0.05 was considered statistically significant.

Results: Most participants were aged 25-34 (406) years and held a bachelor's degree (707). Over half (560) received fertility treatments in the past, with 37.9% (406) having polycystic ovary syndrome (PCOS) and 23.5% (252) with other fertility-impacting medical conditions. Interestingly, 62.1% (665) had not undergone any ART cycles. Of those who did, 51.6% (553) had clinical pregnancies leading to live births. About 23.8% (308) of those with clinical pregnancies faced miscarriages without successful live births. Furthermore, 17.6% (189) reported complications or side effects from past ART procedures, and 31.4% (336) were on ART-related medications or supplements. The linear regression highlighted that individuals with normal weight tended to undergo more ART cycles. However, those with a higher BMI exhibited increased chances of achieving clinical pregnancies and live births.

Conclusion: The study underscores the crucial relationship between BMI and ART efficacy in Saudi women. The data reveals that BMI can significantly influence ART treatment outcomes, especially concerning the number of cycles, clinical pregnancies, and live births. Consequently, BMI should be an essential consideration when evaluating and optimizing the success rates of ART procedures.

## Introduction

Obesity and its implications on health have emerged as significant concerns globally, and Saudi Arabia is no exception. Obesity is known to adversely affect reproductive health, primarily causing infertility through mechanisms such as hormonal imbalances, insulin resistance, chronic inflammation, and compromised ovarian function [1,2]. Assisted reproductive technologies (ART), which encompass revolutionary reproductive medical procedures like in vitro fertilization (IVF) and intracytoplasmic sperm injection (ICSI), present a beacon of hope for infertile couples [3-5]. However, the efficacy of these procedures hinges on several determinants, including the patient's weight, gamete quality, and endometrial receptivity [6].

A stark representation of obesity can be observed in the World Health Organization's (WHO) definitions, categorizing individuals with a body mass index (BMI) of 25 or above as overweight and those with a BMI exceeding 30 as obese [7]. Alarmingly, recent research by Wahabi et al. disclosed that a staggering almost two-thirds of Saudi women, encompassing all age groups and regions, grapple with obesity [8]. This trend warrants grave concern, especially given the known association between obesity and reproductive issues, including menstrual anomalies, anovulation, polycystic ovary syndrome (PCOS), and hindered embryo implantation [9,10].

While several studies have ventured into examining the association between high BMI and ART outcomes, there remains a conspicuous research void concerning Saudi women over the past half-decade. Given the distinct genetic and cultural backdrop of this group, discerning how obesity impinges upon ART success rates becomes pivotal. Grasping this dynamic can pave the way for interventions and strategic planning, refining reproductive outcomes tailored for Saudi women - a move that could resonate profoundly with healthcare professionals and policy framers. This underscores the urgency for more expansive research on this topic, ultimately bolstering reproductive healthcare standards in the region.

In light of the aforementioned premises, this research pivots on a couple of central hypotheses, which will be rigorously evaluated using statistical tools such as correlation analyses, regression, and chi-square tests. Firstly, it is hypothesized that an uptick in BMI might correlate with a downturn in ovarian reserve markers, suggesting the probable adverse influence of high BMI on the volume and caliber of oocytes earmarked for ART [1-3]. Secondly, it's anticipated that women with elevated BMI might record lower clinical pregnancy rates, diminished live birth figures, and escalated miscarriage numbers when juxtaposed against their normal BMI counterparts. This hypothesis insinuates that soaring BMI could potentially attenuate the effectiveness of ART procedures, culminating in suboptimal success rates coupled with augmented obstetric complications [1,2].

Guided by these hypotheses, the primary objective of this cross-sectional exploration is to gauge the impact of heightened BMI on the effectiveness of ART in Saudi women. Delving deeper, the study specifically aspires to discern the correlation between BMI and ovarian reserve metrics in Saudi women and to analyze the nexus between BMI and treatment outcomes - spanning clinical pregnancy rates, live birth statistics, and miscarriage rates-among Saudi women navigating through ART [1-3].

## Materials and methods

Study design and participants

This cross-sectional study was executed from January to August 2023, targeting Saudi women aged 18 years and above who have sought ART procedures, such as IVF or ICSI, for addressing infertility. Of the total of 1071 participants, 155 engaged via an online survey, while 916 responded through hard copy surveys sourced from diverse hospitals in Saudi Arabia.

Ethical approval

The study obtained approval from the Institutional Review Board of King Faisal University, with approval number KFU-REC-2023-AUG-ETHICS1138.

Data collection

All participants completed a comprehensive questionnaire capturing demographic details, medical histories, anthropometric data, ovarian reserve metrics, and treatment results. The data entry process leveraged Google online forms, with the resultant data, subsequently migrated to Microsoft Excel for subsequent analytical processing.

Statistical analysis

A meticulous statistical evaluation was conducted using Statistical Package for the Social Sciences (IBM SPSS Statistics for Windows, IBM Corp., Version 21, Armonk, NY). Descriptive statistics were applied to delineate participant attributes, ART results, and BMI trends. The Chi-square test was employed to probe potential correlations between BMI and participant traits, with p-values underscoring the gravity of these connections. Moreover, a linear regression model was devised to gauge the influence of BMI on diverse ovarian reserve markers, taking into account potential confounders. The significance of associations within this model was ascertained through p-values, with a benchmark p-value of 0.05 denoting statistical significance. Such rigorous analytical methods fortified the interpretation of associations between BMI, participant profiles, and ART results, enhancing the study's reliability.

## Results

Characteristics of study participants

The study comprised a total of 1072 Saudi women who underwent ART. The participants were distributed across different age groups, with the majority falling between 25 and 34 years 406 (37.9%). A significant proportion of participants held a bachelor's degree 707 (66.0%) and were unemployed 560 (52.3%). Most participants resided in the Northern province 630 (58.8%) and lived in urban areas 819 (76.5%) which is displayed in Table 1.

**Table 1:** Characteristics of the study participants Data in the table is represented as N (count) and % (percentage).

		Count	%
Age	18-24	63	5.9%
	25-34	406	37.9%
	35-44	287	26.8%
	45-54	231	21.6%
	55-64	77	7.2%
	65 or more	7	0.7%
Educational Level	bachelor’s degree	707	66.0%
	high school or less	182	17.0%
	diploma	112	10.5%
	doctorate or more	21	2.0%
	master’s degree	49	4.6%
Employment Status	Student	42	3.9%
	Unemployed	560	52.3%
	Retired	63	5.9%
	Employed part-time	28	2.6%
	Employed full-time	378	35.3%
City of Residence	South province	35	3.3%
	Eastern province	21	2.0%
	Northern province	630	58.8%
	Western province	161	15.0%
	Middle province	161	15.0%
	South province	63	5.9%
Geographic Location	Suburban	175	16.3%
	Rural	77	7.2%
	Urban	819	76.5%

Table 2 shows the duration of attempting conception varied among participants, with the highest percentage of 644 (60.1%) having tried for more than two years. Over half of the participants 560 (52.3%) had previously received fertility treatments. Polycystic ovary syndrome (PCOS) was diagnosed in 406 (37.9%) of participants, and 252 (23.5%) had medical conditions affecting fertility. A significant proportion of 210 (19.6%) had undergone weight loss interventions. A substantial number of 427 (39.9%) were taking medications or supplements that might influence fertility. Most participants 672 (62.7%) had not undergone any ART cycles, and 553 (51.6%) had experienced clinical pregnancies resulting in live births.

**Table 2:** General information Data in the table is represented as N (count) and % (percentage). ART: assisted reproductive technology

		Count	%
How long have you been trying to conceive?	less than six months	231	21.6%
	more than two years	644	60.1%
	six months to one year	56	5.2%
	one to two years	140	13.1%
Have you received any previous fertility treatments?	No	511	47.7%
	Yes	560	52.3%
Have you ever been diagnosed with polycystic ovary syndrome (PCOS)?	No	665	62.1%
	Yes	406	37.9%
Have you been diagnosed with any other medical conditions that may affect fertility?	No	819	76.5%
	Yes	252	23.5%
Have you ever undergone any weight loss interventions or programs?	No	861	80.4%
	Yes	210	19.6%
Are you currently taking any medications or supplements that may affect fertility?	No	644	60.1%
	Yes	427	39.9%
How many ART cycles have you undergone so far?	three or more	154	14.4%
	one cycle	140	13.1%
	two cycles	105	9.8%
	None	672	62.7%
Have you experienced any complications or side effects from previous ART treatments?	No	791	73.9%
	Yes	252	23.5%
How would you rate your overall experience with ART treatments?	Very satisfied	196	18.3%
	Satisfied	287	26.8%
	Dissatisfied	91	8.5%
	Neutral	441	41.2%
	Very Dissatisfied	56	5.2%
Have you ever experienced a clinical pregnancy resulting in a live birth?	No	518	48.4%
	Yes	553	51.6%

Gynecological history and overview

Table 3 illustrates the majority of participants had not been diagnosed with hormonal disorders related to fertility 896 (83.7%) or undergone tests to assess ovarian reserve 931 (86.9%). Many participants 882 (82.4%) had not received information about the impact of BMI on ovarian reserve. Additionally, a considerable number of participants 350 (32.7%) experienced irregular menstrual cycles, and 441 (41.2%) had been diagnosed with polycystic ovaries. Symptoms related to hormonal imbalances were reported by 385 (35.9%) of participants, while 294 (27.5%) had thyroid disorders.

**Table 3:** Gynecological history Data in the table is represented as N (count) and % (percentage).

		Count	Column N %
Have you ever been diagnosed with any hormonal disorders related to fertility (e.g., diminished ovarian reserve)?	No	896	83.7%
	Yes	175	16.3%
Have you had any tests to assess your ovarian reserve (e.g., anti-Mullerian hormone, antral)?	No	931	86.9%
	Yes	140	13.1%
Have you received any information from your healthcare provider about the potential impact of BMI on your ovarian reserve?	No	882	82.4%
	Yes	189	17.6%
Are you currently experiencing irregular menstrual cycles?	No	721	67.3%
	Yes	350	32.7%
Have you ever been diagnosed with polycystic ovaries?	No	630	58.8%
	Yes	441	41.2%
Are you currently experiencing any symptoms related to hormonal imbalances (e.g., excessive hair growth, acne)?	No	686	64.1%
	Yes	385	35.9%
Have you ever been diagnosed with any thyroid disorders?	No	777	72.5%
	Yes	294	27.5%

Outcomes for ART and ovarian reserve parameters

In evaluating the outcomes of ART and ovarian reserve parameters, a comprehensive analysis of participant experiences was conducted (Table 4). Among the participants, a significant proportion of 665 (62.1%) had not undergone any ART cycles, while 161 (15.0%) had undergone three or more cycles, indicating varying degrees of engagement with the treatment. In terms of clinical pregnancies, 280 (26.1%) of participants achieved this milestone, whereas 756 (70.6%) did not. Further scrutinizing clinical pregnancies, it was observed that 294 (27.5%) of these cases resulted in live births, with the remaining 672 (62.7%) not culminating in successful live births. In instances where clinical pregnancies did not lead to live births, 308 (23.8%) reported experiencing miscarriages, pointing to the complexities of reproductive outcomes. Notably, a subset of participants 189 (17.6%) encountered complications or side effects arising from previous ART treatments, highlighting the multifaceted nature of these interventions. Moreover, a distinct group of 196 (18.3%) underwent supplementary procedures during their ART journey. In terms of medication intake, 336 (31.4%) of participants were actively taking medications or supplements tailored to their ART regimen. Importantly, 259 (24.2%) of participants had been informed by healthcare providers about the potential influence of BMI on treatment outcomes, showcasing the relevance of BMI in reproductive health conversations. Additionally, 217 (20.3%) of participants received counseling or support concerning weight management during their ART treatment, underscoring the holistic nature of fertility care.

**Table 4:** Outcomes for ART and ovarian reserve parameters Data in the table is represented as N (count) and % (percentage). ART: assisted reproductive technology

		Count	Column N %
How many ART cycles have you undergone so far?	Three or more	161	15.0%
	Two cycles	126	11.8%
	One cycle	119	11.1%
	None	665	62.1%
In your most recent ART cycle, did you achieve a clinical pregnancy?	No	756	70.6%
	Yes	280	26.1%
If yes, did the clinical pregnancy result in a live birth?	No	672	62.7%
	Yes	294	27.5%
If no, did you experience a miscarriage?	No	700	65.4%
	Yes	308	23.8%
Have you experienced any complications or side effects from previous ART treatments?	No	882	82.4%
	Yes	189	17.6%
Have you undergone any additional procedures or interventions (e.g., egg or embryo freezing, preimplantation genetic testing) during your ART treatment?	No	875	81.7%
	Yes	196	18.3%
Are you currently taking any medications or supplements prescribed for your ART treatment?	No	735	68.6%
	Yes	336	31.4%
Have you been informed by your healthcare provider about the potential impact of BMI on treatment outcomes?	No	812	75.8%
	Yes	259	24.2%
Have you received any counseling or support related to weight management during your ART treatment?	No	854	79.7%
	Yes	217	20.3%

BMI

Table 5 within the context of BMI and its associations, a meticulous examination of BMI distribution and its implications on ART outcomes was performed. The majority of participants fell within the normal weight range of 752 (70.2%), while 187 (17.5%) were classified as overweight, and 132 (12.3%) were underweight.

**Table 5:** Body mass index Data in the table is represented as N (count) and % (percentage).

	Frequency	%
Normal	752	70.2
Overweight	187	17.5
Underweight	132	12.3

Association between BMI and participant's characteristics

Table 6 dives into the connection between participants' BMI and their demographic characteristics. The distribution of BMI categories, including normal, overweight, and underweight, is examined across various participant attributes. While no significant associations were detected between BMI and these characteristics, this analysis provides valuable insight into the interplay between BMI and demographics, highlighting the complexity of factors influencing reproductive health.

**Table 6:** Association between BMI and participant's characteristics Data in the table is represented as N (count) and % (percentage). A p-value of <0.05 was considered statistically significant. BMI: body mass index

		BMI						P-value
		Normal		Overweight		Underweight		
		Count	%	Count	%	Count	%	
Age	18-24	42	66.7%	15	23.8%	6	9.5%	0.746
	25-34	284	70.0%	79	19.5%	43	10.6%	
	35-44	202	70.4%	47	16.4%	38	13.2%	
	45-54	164	71.0%	34	14.7%	33	14.3%	
	55-64	55	71.4%	11	14.3%	11	14.3%	
	65 or more	5	71.4%	1	14.3%	1	14.3%	
Educational Level	bachelor’s degree	498	70.4%	119	16.8%	90	12.7%	0.950
	high school or less	126	69.2%	34	18.7%	22	12.1%	
	diploma	79	70.5%	21	18.8%	12	10.7%	
	doctorate or more	14	66.7%	4	19.0%	3	14.3%	
	master’s degree	35	71.4%	9	18.4%	5	10.2%	
Employment Status	Student	30	71.4%	7	16.7%	5	11.9%	0.977
	Unemployed	390	69.6%	106	18.9%	64	11.4%	
	Retired	45	71.4%	9	14.3%	9	14.3%	
	Employed part-time	20	71.4%	5	17.9%	3	10.7%	
	Employed full-time	267	70.6%	60	15.9%	51	13.5%	
City of Residence	South province	25	71.4%	7	20.0%	3	8.6%	0.554
	Eastern province	14	66.7%	4	19.0%	3	14.3%	
	Northern province	450	71.4%	94	14.9%	86	13.7%	
	Western province	110	68.3%	34	21.1%	17	10.6%	
	Middle province	111	68.9%	34	21.1%	16	9.9%	
	South province	42	66.7%	14	22.2%	7	11.1%	
Geographic Location	Suburban	125	71.4%	28	16.0%	22	12.6%	0.964
	Rural	55	71.4%	12	15.6%	10	13.0%	
	Urban	572	69.8%	147	17.9%	100	12.2%	

Table 7 shows the relationship between BMI and ovarian reserve parameters was explored. A significant association was found between BMI and the number of ART cycles undergone (p < 0.001), where participants with higher BMI tended to have undergone fewer cycles. Similarly, BMI was significantly associated with the achievement of clinical pregnancy (p < 0.001) and live births resulting from clinical pregnancies (p < 0.001), with higher BMI associated with lower rates of success. Moreover, the occurrence of miscarriages was also significantly associated with BMI (p < 0.001).

**Table 7:** Association between BMI and ovarian reserve parameters Data in the table is represented as N (count) and % (percentage). A p-value of <0.05 was considered statistically significant. ART: assisted reproductive technology, BMI: body mass index

		BMI						P-value
		Normal		Overweight		Underweight		
		Count	Row N %	Count	Row N %	Count	Row N %	
How many ART cycles have you undergone so far?	Three or more	116	72.0%	27	16.8%	18	11.2%	<0.001
	Two cycles	89	70.6%	27	21.4%	10	7.9%	
	One cycle	82	68.9%	22	18.5%	15	12.6%	
	None	465	69.9%	111	16.7%	89	13.4%	
In your most recent ART cycle, did you achieve a clinical pregnancy?	No	528	69.8%	132	17.5%	96	12.7%	<0.001
	Yes	199	71.1%	49	17.5%	32	11.4%	
If yes, did the clinical pregnancy result in a live birth?	No	471	70.1%	120	17.9%	81	12.1%	<0.001
	Yes	206	70.1%	49	16.7%	39	13.3%	
If not, did you experience a miscarriage?	No	491	70.1%	122	17.4%	87	12.4%	<0.001
	Yes	216	70.1%	55	17.9%	37	12.0%	

Linear regression model between BMI and ovarian reserve parameters

Table 8 represents a linear regression model that was constructed to further investigate the relationship between BMI and ovarian reserve parameters. After controlling for potential confounders, the odds of undergoing a greater number of ART cycles were significantly higher in normal-weight participants (Exp[B] = 1.004, 95% CI = 1.000-1.113, p = 0.007). Similarly, the odds of achieving clinical pregnancy increased with higher BMI (Exp[B] = 1.034, 95% CI = 1.000-1.302, p = 0.010). Participants with higher BMI also had increased odds of a clinical pregnancy resulting in a live birth (Exp[B] = 1.015, 95% CI = 1.000-1.151, p = 0.030). Notably, the odds of experiencing a miscarriage were significantly higher for participants with underweight BMI (Exp[B] = 1.910, 95% CI = 1.000-13.719, p = 0.020).

**Table 8:** Linear regression model between BMI and ovarian reserve parameters Data in the table is represented as N (count) and % (percentage). A p-value of <0.05 was considered statistically significant. ART: assisted reproductive technology, BMI: body mass index

Predictors	Exp(B), 95% CI	P-value
How many ART cycles have you undergone so far?	1.004 (1-1.113)	0.007
In your most recent ART cycle, did you achieve a clinical pregnancy?	1.034 (1-1.302)	0.01
If yes, did the clinical pregnancy result in a live birth?	1.015 (1-1.151)	0.03
If not, did you experience a miscarriage?	1.91 (1-13.719)	0.02

## Discussion

This research delves into the intricate relationship between BMI and the efficacy of ART in Saudi women, an area underscoring the broader implications of obesity on reproductive health as introduced previously [1]. Rooted in a methodological approach that segmented participants based on BMI - high BMI (≥30 kg/m²) and normal BMI (18.5-24.9 kg/m²) - the outcomes produced from this study lend credence to existing literature and provide fresh avenues for clinical and research applications.

A significant proportion of participants belonged to the age bracket of 20-40 years, and the majority underwent ART interventions, including IVF or ICSI, to combat infertility. Data extraction methodologies involved both on-site and electronic self-administered questionnaires, ensuring a comprehensive approach [2]. This method was justified as it offered a holistic understanding of demographic nuances, medical antecedents, ovarian metrics, and treatment results.

In assessing the study's outcomes, a pronounced relationship between BMI and varying ovarian reserve markers was identified [3]. This directly emphasizes that BMI is instrumental in forecasting the success rate of ART endeavors. Notably, a rise in BMI correlated with fewer ART cycles, diminished instances of clinical pregnancies and live births, and a heightened probability of miscarriages. These observations resonate with extant research that documented the detrimental consequences of elevated weight on fertility [4,5].

Contrarily, a surprising outcome noted higher likelihoods of clinical pregnancies culminating in live births amongst high BMI participants. This seemingly counteractive result might be attributed to the intricate balance between BMI, hormonal shifts, and reproductive dynamics in individuals with obesity. Enhanced estrogen synthesis by fat tissues might facilitate better implantation probabilities and subsequent pregnancy outcomes [6,7]. Yet, the exact mechanisms necessitate deeper probing in subsequent studies.

Equally worth noting is the established link between underweight BMI categorizations and escalated miscarriage rates, reaffirming previous literature insights [8,9]. Hormonal irregularities and diminished ovarian functionality in individuals with lower weight might be potential causatives for the increased miscarriage predisposition. These results underscore the significance of a balanced BMI for optimal fertility outcomes.

While this research didn't discern evident links between BMI and demographic attributes, it remains paramount to underline that factors like age, academic qualifications, and economic standing could modulate both BMI and reproductive wellness [10-13].

In conclusion, this study's findings accentuate the clinical ramifications of BMI on ART. Medical practitioners should be apprised of BMI's salience when evaluating patients for ART [14]. Advising patients on BMI's conceivable influence on treatment results and championing a balanced lifestyle, inclusive of weight management, could augment the likelihood of triumphant ART outcomes. However, the study is not without its limitations. The potential influence of socio-demographic variables on the relationship between BMI and ART outcomes warrants a deeper dive in subsequent research.

## Conclusions

The interplay between BMI and the success rates of ART in Saudi women is both intricate and profound. This study reinforces the pivotal role BMI plays in determining the efficacy of ART treatments, with both higher and lower BMIs showcasing potential impacts on fertility outcomes. The counterintuitive finding of increased successful pregnancies in women with higher BMIs signals the need for further research to unravel the underlying mechanisms. Moreover, the importance of maintaining an optimal BMI for enhanced reproductive success cannot be overstated. Medical professionals should be cognizant of this relationship, ensuring that patient assessments and counseling incorporate weight management and its ramifications on ART. Future studies might also delve deeper into the socio-demographic factors and their potential influence on this BMI-ART relationship to provide a holistic perspective and guide clinical decisions.

## Appendices

Table 9 displays the questionnaire used in the research.

**Table 9:** Questionnaire

Questionnaire:
Section 1: Demographics
Age:
Under 18
18-24
25-34
35-44
45-54
55-64
Above 65
Gender:
Male
Female
Education level:
High school or less
Diploma
Bachelor's degree
Master's degree
Doctorate or higher
Employment Status:
a. Employed full-time
b. Employed part-time
c. Unemployed
d. Student
e. Retired
Other
City of residence:
Middle Province
Eastern Province
Northern Province
South Province
Western Province
Others
Geographic Location:
a. Urban
b. Suburban
c. Rural
Section 1: General Questions
How would you rate the severity of the pediatric traumatic brain injury you have encountered?
a) Mild
b) Moderate
c) Severe
What imaging techniques were utilized for the evaluation of pediatric traumatic brain injury? (Select all that apply)
a) Computed Tomography (CT) scan
b) Magnetic Resonance Imaging (MRI)
c) Other (Please specify: ________________)
Were the imaging findings consistent with the clinical presentation?
a) Yes
b) No
c) Not sure
How did the imaging findings influence the clinical decision-making process?
a) They confirmed the diagnosis and guided treatment decisions.
b) They provided additional information but did not significantly impact the clinical management.
c) They were inconclusive or did not correlate with the clinical presentation.
Were surgical interventions required based on the imaging findings?
a) Yes
b) No
c) Not applicable
How satisfied were you with the diagnostic accuracy of the imaging techniques used?
a) Very satisfied
b) Moderately satisfied
c) Not satisfied
Did the imaging techniques provide valuable prognostic information?
a) Yes
b) No
c) Not sure
How frequently do you encounter challenges related to the availability of MRI for pediatric TBI cases?
a) Frequently
b) Occasionally
c) Rarely
Are you concerned about the radiation exposure associated with CT scans in pediatric patients?
a) Yes, I am very concerned.
b) Somewhat concerned.
c) No, I am not concerned.
Do you believe that there is a need for evidence-based guidelines regarding the appropriate use of imaging techniques in pediatric TBI?
a) Yes.
b) Maybe it could be helpful.
c) No, I don't think it is necessary.
Section 2: Hypothesis 1 - Association between abnormal CT scan findings and the presence of pediatric TBI
Did the CT scan findings reveal any abnormal intracranial findings suggestive of pediatric TBI?
a) Yes
b) No
c) Not sure
If abnormal intracranial findings were present on the CT scan, what was the nature of the injury? (Select all that apply)
a) Skull fracture
b) Epidural hematoma
c) Subdural hematoma
d) Subarachnoid hemorrhage
e) Intracerebral hemorrhage
f) Other (Please specify: ________________)
Were there any discrepancies between the CT scan findings and the clinical presentation?
a) Yes
b) No
c) Not sure
Did the presence of abnormal CT scan findings correlate with the severity of pediatric TBI?
a) Yes, strongly correlated
b) Yes, moderately correlated
c) No correlation was observed
Did the abnormal CT scan findings impact the choice of treatment options for pediatric TBI?
a) Yes
b) No
c) Not applicable
Were any further confirmatory tests or additional imaging studies performed after the initial CT scan?
a) Yes
b) No
c) Not applicable
How confident were you in the accuracy of the CT scan findings?
a) Very confident
b) Moderately confident
c) Not confident
Did the CT scan findings significantly contribute to the clinical management decisions?
a) Yes, significantly
b) Yes, to some extent
c) No, they had minimal impact
Were there any cases where the CT scan findings did not align with the clinical outcomes?
a) Yes
b) No
c) Not sure
In your experience, what is the overall reliability of CT scans in detecting pediatric TBI?
a) Very reliable
b) Moderately reliable
c) Not Reliable
Section 3: Hypothesis 2 - Correlation between specific MRI characteristics and the severity of pediatric TBI
Did the MRI findings reveal any specific characteristics indicative of pediatric TBI?
a) Yes
b) No
c) Not sure
If specific characteristics were present on the MRI, what was observed? (Select all that apply)
a) Diffuse axonal injury
b) Intracranial hemorrhage
c) Contusion(s)
d) Edema
e) Other (Please specify: ________________)
Were the MRI findings consistent with the severity of pediatric TBI?
a) Yes, strongly consistent
b) Yes, moderately consistent
c) No consistency observed
Did the specific MRI characteristics influence the choice of treatment options for pediatric TBI?
a) Yes
b) No
c) Not applicable
Did the presence and extent of specific MRI characteristics correlate with the prognosis of pediatric TBI?
a) Yes, strongly correlated
b) Yes, moderately correlated
c) No correlation was observed
Were there any discrepancies between the MRI findings and the clinical presentation?
a) Yes
b) No
c) Not sure
Did the MRI findings contribute to a better understanding of the anatomical involvement in pediatric TBI cases?
a) Yes, significantly
b) Yes, to some extent
c) No, they had minimal impact
How confident were you in the accuracy of the MRI findings?
a) Very confident
b) Moderately confident
c) Not confident
Were there any cases where the MRI findings did not align with the clinical outcomes?
a) Yes
b) No
c) Not sure
In your experience, what is the overall reliability of MRI in detecting and characterizing pediatric TBI?
a) Very reliable
b) Moderately reliable
c) Not Reliable
Q

## References

[1] Yong W, Wang J, Leng Y, Li L, Wang H (2023). Role of obesity in female reproduction. Int J Med Sci.

[2] Pasquali R, Patton L, Gambineri A (2007). Obesity and infertility. Curr Opin Endocrinol Diabetes Obes.

[3] Dornelles VC, Hentschke MR, Badalotti M (2022). The impact of body mass index on laboratory, clinical outcomes and treatment costs in assisted reproduction: a retrospective cohort study. BMC Womens Health.

[4] Gunby J, Daya S (2006). Assisted reproductive technologies (ART) in Canada: 2002 results from the Canadian ART Register. Fertil Steril.

[5] Rafique M, Al-Badr A, Saleh A, Al-Jaroudi DH (2021). Economic perspective of evaluating fertility treatment in obese and overweight infertile women. Saudi Med J.

[6] (2000). Obesity: preventing and managing the global epidemic: report of a WHO consultation. https://apps.who.int/iris/handle/10665/42330.

[7] Wahabi H, Fayed AA, Shata Z (2022). The impact of age, gender, temporality, and geographical region on the prevalence of obesity and overweight in Saudi Arabia: scope of evidence. Healthcare.

[8] Maheshwari A, Stofberg L, Bhattacharya S (2007). Effect of overweight and obesity on assisted reproductive technology - a systematic review. Hum Reprod Update.

[9] Al-Hazzaa Al-Hazzaa, Hazzaa M (2019). Physical activity and sedentary behaviors in bariatric surgery patients: a scoping review. Saudi J Obes.

[10] Hu D, Huang B, Xiong M (2022). Impact of elevated body mass index on cumulative live birth rate and obstetric safety in women undergoing assisted reproductive technology. Sci Rep.

[11] Purewal S, Chapman SC, van den Akker OB (2019). A systematic review and meta-analysis of lifestyle and body mass index predictors of successful assisted reproductive technologies. J Psychosom Obstet Gynaecol.

[12] Bellver J (2022). BMI and miscarriage after IVF. Curr Opin Obstet Gynecol.

[13] Banker M, Sorathiya D, Shah S (2017). Effect of body mass index on the outcome of in-vitro fertilization/intracytoplasmic sperm injection in women. J Hum Reprod Sci.

[14] Elnashar AM (2023). Update on obesity and assisted reproductive technology. Middle East Fertil Soc J.

